# Supplementary material for: An isotropic zero thermal expansion alloy with super-high toughness
Source: Nat Commun. 2024 Mar 13;15:2252. doi: 10.1038/s41467-024-46613-0 (PMC10937970; doi:10.1038/s41467-024-46613-0)
Supplement: Supplementary file 1 — Supplementary Information [file 41467_2024_46613_MOESM1_ESM.pdf]

SUPPORTING INFORMATION

An isotropic zero thermal expansion alloy with super-high toughness

Chengyi Yu[1], Kun Lin[1], Qinghua Zhang[2], Huihui Zhu[1], Ke An[3], Yan Chen[3], Dunji Yu[3], Tianyi Li[4], Xiaoqian Fu[5], Qian Yu,[5] Li You[1], Xiaojun Kuang[6], Yili Cao[1], Qiang Li[1], Jinxia Deng[1] and Xianran Xing[1,*]

[1] Beijing Advanced Innovation Center for Materials Genome Engineering, and Institute of Solid State Chemistry, University of Science and Technology Beijing, Beijing 100083, China.

[2] Institution of Physics, Chinese Academic of Science, No.8, 3rd South Street, Zhongguancun, Haidian District, Beijing, 100190 P. R. China.

[3] Neutron Scattering Division, Oak Ridge National Laboratory, Oak Ridge, TN, USA.

[4] X-Ray Science Division, Argonne National Laboratory, Argonne, IL 60439, USA

[5] Center of Electron Microscopy and State Key Laboratory of Silicon Materials, Department of Materials Science and Engineering, Zhejiang University, Hangzhou, China.

[6] Guangxi Key Laboratory of Electrochemical and Magnetochemical Functional Materials, College of Chemistry and Bioengineering, Guilin University of Technology, Guilin 541004, P. R. China.

*Corresponding author. Email: *xing@ustb.edu.cn*

Contents

Supplementary Tables 3

Supplementary Table 1 | The chemical composition of elements of the $LaFe_{43.5+x}Si_{2.6+0.06x}$ (x = 0, 4, 8, 12, 16) series alloys which were attained by EPMA. 3

Supplementary Table 2 | The chemical composition of elements of the $LaFe_{0.939x}Co_{0.061x}Si_{0.0583x}$ (x = 37.5, 47.5, 57. and 67.5, labeled as S-1, S-2, S-3, and S-4, respectively) series alloys which were attained by EPMA. 4

Supplementary Table 3 | The summary of ultimate strength (δ_{US}, MPa) and strain (ε_f, %) in typical ZTE materials. 5

Supplementary Figures 6

Supplementary Fig. 1 | The calculation of Si_L/Si_α and Fe_L/Fe_α partition coefficient in La-Fe-Si ternary system. 6

Supplementary Fig. 2 | The calculation of Si_L/Si_α, Fe_L/Fe_α and Co_L/Co_α partition coefficient. 7

Supplementary Fig. 3 | Schematic diagram of the relationship between ingot and coordinate system. 8

Supplementary Fig. 4 | The cyclic thermal shock experiment undergoes a thermal shock from 77 K to 373 K. 9

Supplementary Fig. 5 | The Rietveld refinement of synchrotron X-ray diffraction profile of the as-cast samples in composition $LaFe_{0.939x}Co_{0.061x}Si_{0.0583x}$ (x = 37.5, 47.5, 57.5, and 67.5, labeled as S-1, S-2, S-3, and S-4, respectively). 10

Supplementary Fig. 6 | The typical diffraction (*hkl*) reflections of the dual-phase alloy. 11

Supplementary Fig. 7 | The microstructure and inverse pole figure (IPFZ) of S-3 alloy. 12

Supplementary Fig. 8 | The electro-probe micro-analyzer (EPMA) mappings of the S-3. 13

Supplementary Fig. 9 | The interface structure of the S-3alloy. 14

Supplementary Fig. 10 | Mechanical performance and small parts. 15

Supplementary Fig. 11 | Schematic diagram of the in-situ neutron diffraction experimental set-up from the top view. 16

Supplementary Fig. 12 | The compressive stress-strain curves of the pure L phase ($LaFe_{10.30}Co_{0.83}Si_{1.87}$) and α phase ($Fe_{92.42}Co_{4.34}Si_{3.07}$). 17

Supplementary Fig. 13 | Microstructure evolution of the dual-phase alloy (ZTE composition). 18

Supplementary Fig. 14 | Microstructure evolution of the dual-phase alloy (ZTE composition). 19

Supplementary Fig. 15 | The evolution of stacking fault illustrated by the crystal structure. 20

Supplementary Fig. 16 | Dual-phase interface structures at 15% strain (ε). 21

Supplementary Fig. 17 | Fracture morphology of the S-3 and pure $La(Fe, Co, Si)_{13}$ compound, dense and cross stripes demonstrated the large plastic deformation before failure. 22

Supplementary Fig. 18 | A typical interface of the dual-phase alloy (ZTE composition) at 15 % strain (ε). 23

References 24

Supplementary Tables

Supplementary Table 1 | The chemical composition of elements of the $LaFe_{43.5+x}Si_{2.6+0.06x}$ (x = 0, 4, 8, 12, 16) series alloys which were attained by EPMA.

Compositions	α Phase, elements atom%			L Phase, elements atom%			Partition coefficient	
	Si	Fe	La	Si	Fe	La	$Si_L/Si_α$	$Fe_L/Fe_α$
$LaFe_{43.5}Si_{2.6}$	3.162	96.733	0.104	11.731	82.457	5.812	3.709	0.852 ①
	3.159	96.649	0.191	11.834	82.398	5.769	3.746	0.852 ②
	3.622	96.285	0.093	12.022	82.343	5.6345	3.319	0.855 ③
$LaFe_{47.5}Si_{2.84}$	3.158	96.721	0.121	11.953	82.206	5.505	3.785	0.850 ①
	3.121	96.719	0.159	11.980	82.206	5.484	3.839	0.850 ②
	3.274	96.548	0.176	11.844	82.354	5.470	3.617	0.853 ③
$LaFe_{51.5}Si_{3.08}$	3.719	96.279	0.055	12.099	87.170	6.035	3.253	0.905 ①
	3.737	96.260	0.072	12.264	86.989	6.091	3.282	0.904 ②
	3.766	96.231	0.090	11.972	87.304	6.049	3.180	0.907 ③
$LaFe_{55.5}Si_{3.32}$	4.204	95.132	0.663	12.940	81.403	5.656	3.078	0.856 ①
	4.172	95.714	0.113	13.159	81.181	5.658	3.154	0.848 ②
	4.311	95.570	0.119	12.512	81.769	5.719	2.902	0.856 ③
$LaFe_{59.5}Si_{3.56}$	4.104	95.819	0.077	12.743	81.562	5.695	3.105	0.851 ①
	4.043	95.855	0.101	12.332	82.013	5.654	3.050	0.856 ②
	3.927	96.023	0.049	12.212	82.213	5.575	3.109	0.856 ③
Average ratio $Si_L/Si_α$: 3.342; $Fe_L/Fe_α$: 0.863								

Note：①、②、③ represent that three separate area collections were selected for each sample.

Supplementary Table 2 | The chemical composition of elements of the $LaFe_{0.939x}Co_{0.061x}Si_{0.0583x}$ (x = 37.5, 47.5, 57. and 67.5, labeled as S-1, S-2, S-3, and S-4, respectively) series alloys which were attained by EPMA.

Compositions	α Phase, elements atom%				L Phase, elements atom%				Partition coefficient			
	Co	Si	Fe	La	Co	Si	Fe	La	$Si_L/Si_α$	$Fe_L/Fe_α$	$Co_L/Co_α$	
S-1	5.281	2.383	92.256	0.080	6.699	10.116	77.493	5.692	4.245	0.840	1.268	①
	5.277	2.476	92.157	0.090	6.639	10.089	77.904	5.368	4.075	0.845	1.258	②
	5.348	2.496	92.024	0.133	6.828	10.416	77.004	5.752	4.173	0.837	1.277	③
S-2	5.431	3.064	91.367	0.137	6.854	11.547	76.009	5.591	3.769	0.832	1.262	①
	5.452	3.140	91.256	0.152	6.981	11.517	75.853	5.65	3.668	0.831	1.280	②
	5.499	3.053	91.288	0.160	6.833	10.933	76.932	5.302	3.581	0.843	1.243	③
S-3	5.452	3.390	91.064	0.094	6.956	11.624	75.764	5.656	3.429	0.832	1.276	①
	5.505	3.307	91.100	0.088	6.682	11.019	78.044	4.255	3.332	0.857	1.214	②
	5.519	3.423	90.965	0.093	6.915	11.862	75.989	5.234	3.465	0.835	1.253	③
S-4	5.616	3.535	90.752	0.097	7.196	12.189	74.723	5.891	3.448	0.823	1.281	①
	5.626	3.551	90.759	0.064	7.047	12.428	74.994	5.531	3.500	0.826	1.253	②
	5.621	3.515	90.800	0.064	7.131	12.020	75.286	5.562	3.420	0.829	1.269	③

Average ratio $Si_L/Si_α$: 3.675; $Fe_L/Fe_α$: 0.836; $Co_L/Co_α$: 1.261

Note：①、②、③ represent that three separate area collections were selected for each sample.

Supplementary Table 3 | The summary of ultimate strength (δ_{US}, MPa) and strain (ε_f, %) in typical ZTE materials.

Typical ZTE materials	Strain (ε_f, %)	Ultimate strength (δ_{US}, MPa)	References
$TbCo_{1.9}Fe_{0.1}$	1.5	260	1
$Ho_2Fe_{16}Cr$	2.0	50	2
MnCoGe	1.0	70.4	3
$LaFe_{11}Si_2H_x$	1.0	60	4
$Mn_3Cu_{0.5}Ge_{0.5}N$	1.0	161	5
$Hf_{0.87}Ta_{0.13}Fe_2$	2.7	380	6
$Mn_3Ge_{0.9}Sn_{0.1}$	5.0	450	7
Mn_3Ge	5.6	204	7
$Ho_{0.07}Fe_{0.93}$	5.68	1275.8	8
$Ho_{0.05}Fe_{0.95}$	8.08	796.8	8
$Ho_{0.04}Fe_{0.96}$	15.6	878	8
GDFC	1.2	154	9
$LaFe_{14.2}Co_{1.2}Si_{1.6}$	3.2	861.1	10
$LaFe_{16.2}Co_{1.2}Si_{1.6}$	3.6	883.6	10
$LaFe_{18.2}Co_{1.2}Si_{1.6}$	3.78	968.8	10
$LaFe_{20.2}Co_{1.2}Si_{1.6}$	3.87	1001.6	10
$LaFe_{10.1}Cu_{0.5}Si_{2.4}$	2.8	1325	11
$LaFe_{10.2}Cu_{0.4}Si_{2.4}$	2.6	1240	11
$La(Fe, Co, Si)_{13}/Cu_{54.6}$	12.6	441.1	12
$La(Fe, Co, Si)_{13}/Cu_{39.7}$	6.4	360.6	12
$La(Fe, Co, Si)_{13}/Cu_{25.6}$	1.9	218.5	12
$La(Fe, Si)_{13}/Resin_3$	4.2	116.2	13
$La(Fe, Si)_{13}/Resin_{20}$	4.1	180.9	13
$La(Fe, Si)_{13}/Resin_{80}$	5.3	203.8	13
$Ho_2Fe_{16}Co$	2	49	14
Er-Fe-V-Mo	2.31	1631.4	15
$La(Fe, Co, Si)_{13}$	1.6	256.4	16
S-3	30.9	1110.1	This work
Invar	30	680.0	ε = 30 %

Supplementary Figures

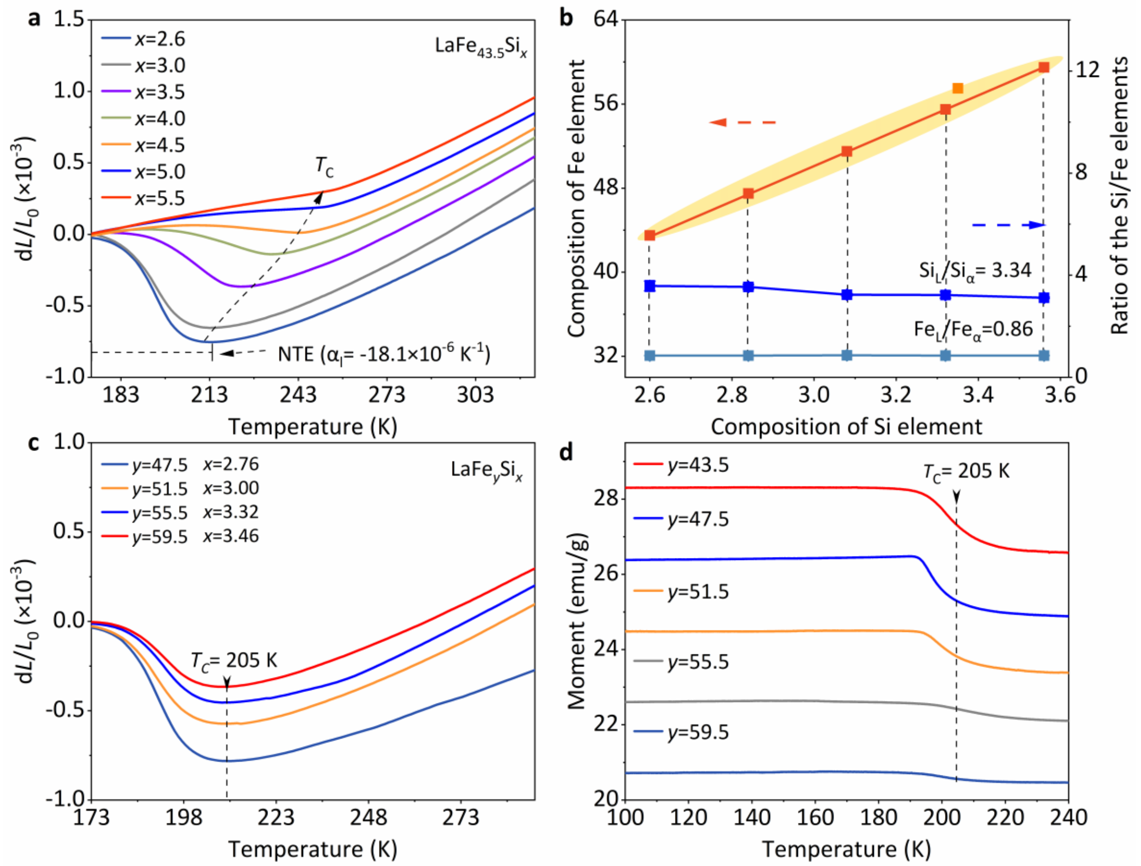

Supplementary Fig. 1 | The calculation of Si_L/Si_α and Fe_L/Fe_α partition coefficient in La-Fe-Si ternary system. a, The dilatometer thermal expansion of series $LaFe_{43.5}Si_x$ (x = 2.6, 3.0, 3.5, 4.0, 4.5, 5.0, 5.5) alloys. **b**, The ratio of Si_L/Si_α and Fe_L/Fe_α in response to different compositions. **c**, The dilatometer thermal expansion of series $LaFe_{43.5+x}Si_{2.6+0.06x}$ (x = 0, 4, 8, 12, 16) alloys. **d**, The corresponding magnetic measurements of the series alloys.

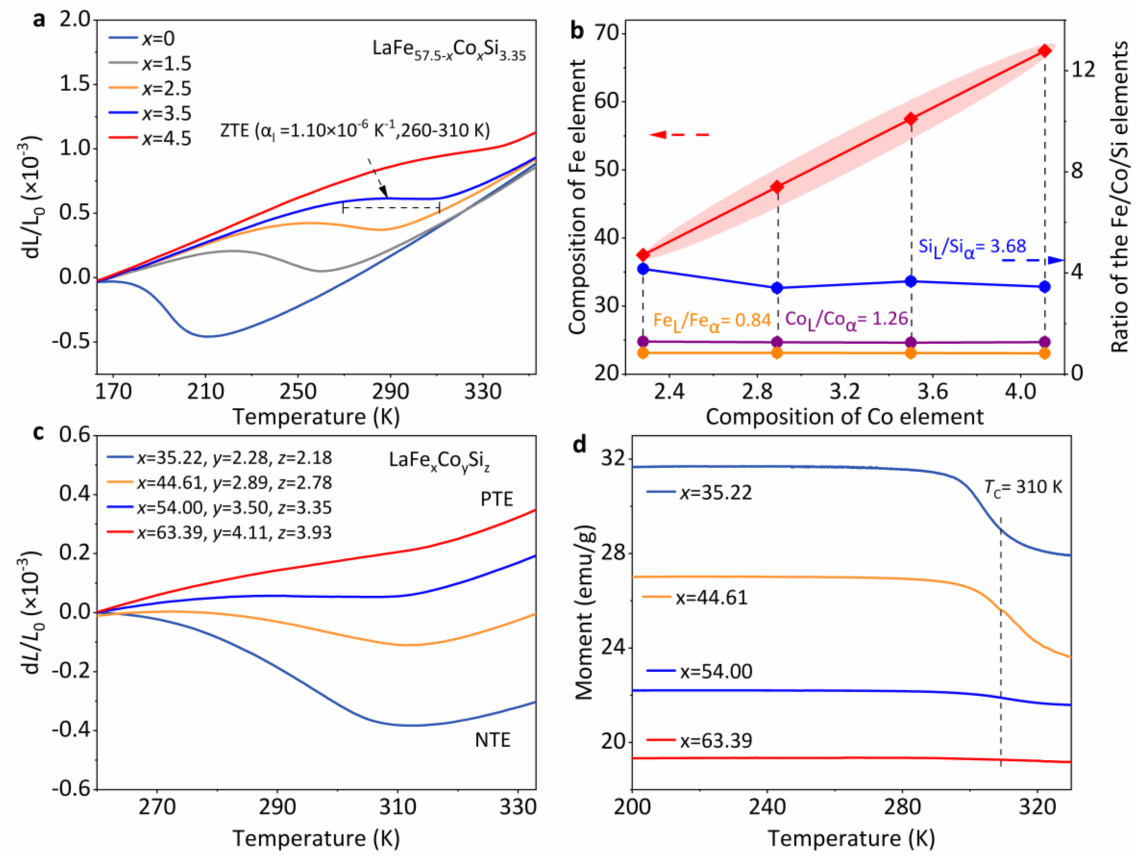

Supplementary Fig. 2 | The calculation of Si_L/Si_α, Fe_L/Fe_α and Co_L/Co_α partition coefficient. **a**, The dilatometer thermal expansion of series $LaFe_{57.5\text{-}x}Co_xSi_{3.35}$ (x = 0, 1.5, 2.5, 3.5, 4.5) alloys. **b**, The ratio of Si_L/Si_α, Fe_L/Fe_α, and Co_L/Co_α in response to different compositions. **c**, The dilatometer thermal expansion of series $LaFe_{57.5\text{-}x}Co_xSi_{3.35}$ (x = 0, 1.5, 2.5, 3.5, 4.5) alloys. **d**, The corresponding magnetic measurements of the series alloys.

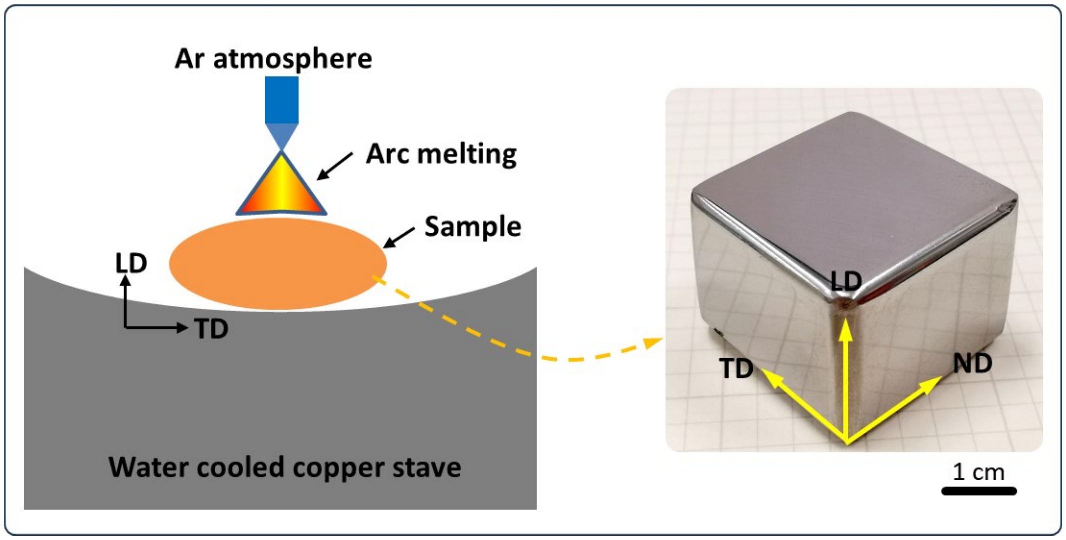

Supplementary Fig. 3 | Schematic diagram of the relationship between ingot and coordinate system. The temperature gradient along the LD direction is the reason for the preferential growth of the sample.

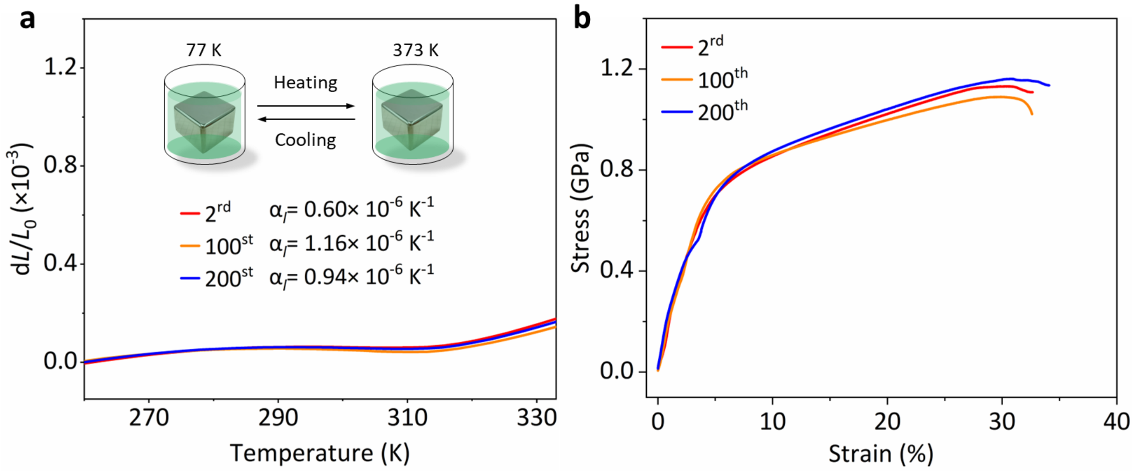

Supplementary Fig. 4 | The cyclic thermal shock experiment undergoes a thermal shock from 77 K to 373 K. a, The dilatometer thermal expansions of S-3 alloy in the 1st, 100th, and 200th cycles. **b**, The compressive stress-strain curves of the S-3 alloy after the 1st, 100th, and 200th cycles.

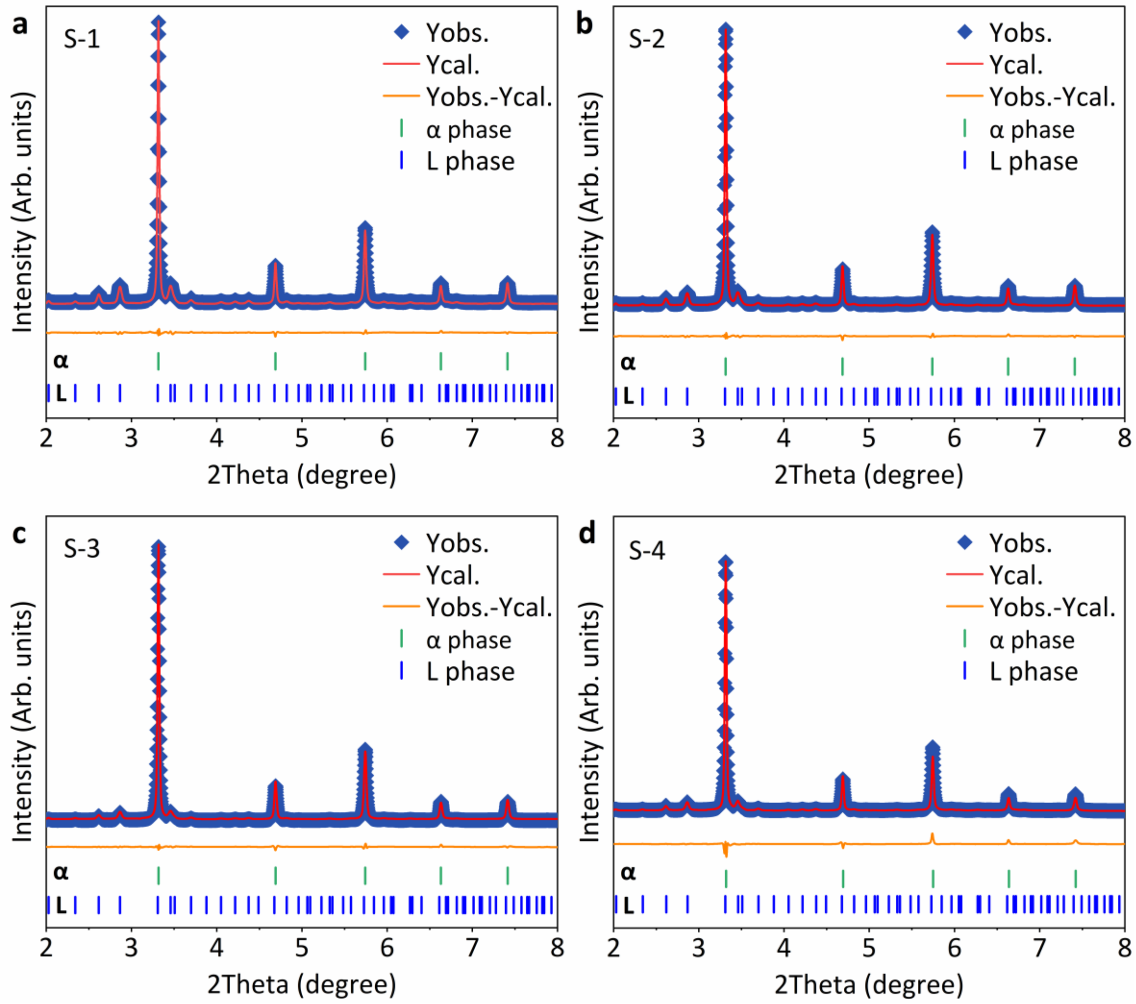

Supplementary Fig. 5 | a-d, The Rietveld refinement of synchrotron X-ray diffraction profile of the as-cast samples in composition $LaFe_{0.939x}Co_{0.061x}Si_{0.0583x}$ (x = 37.5, 47.5, 57.5, and 67.5, labeled as S-1, S-2, S-3, and S-4, respectively).

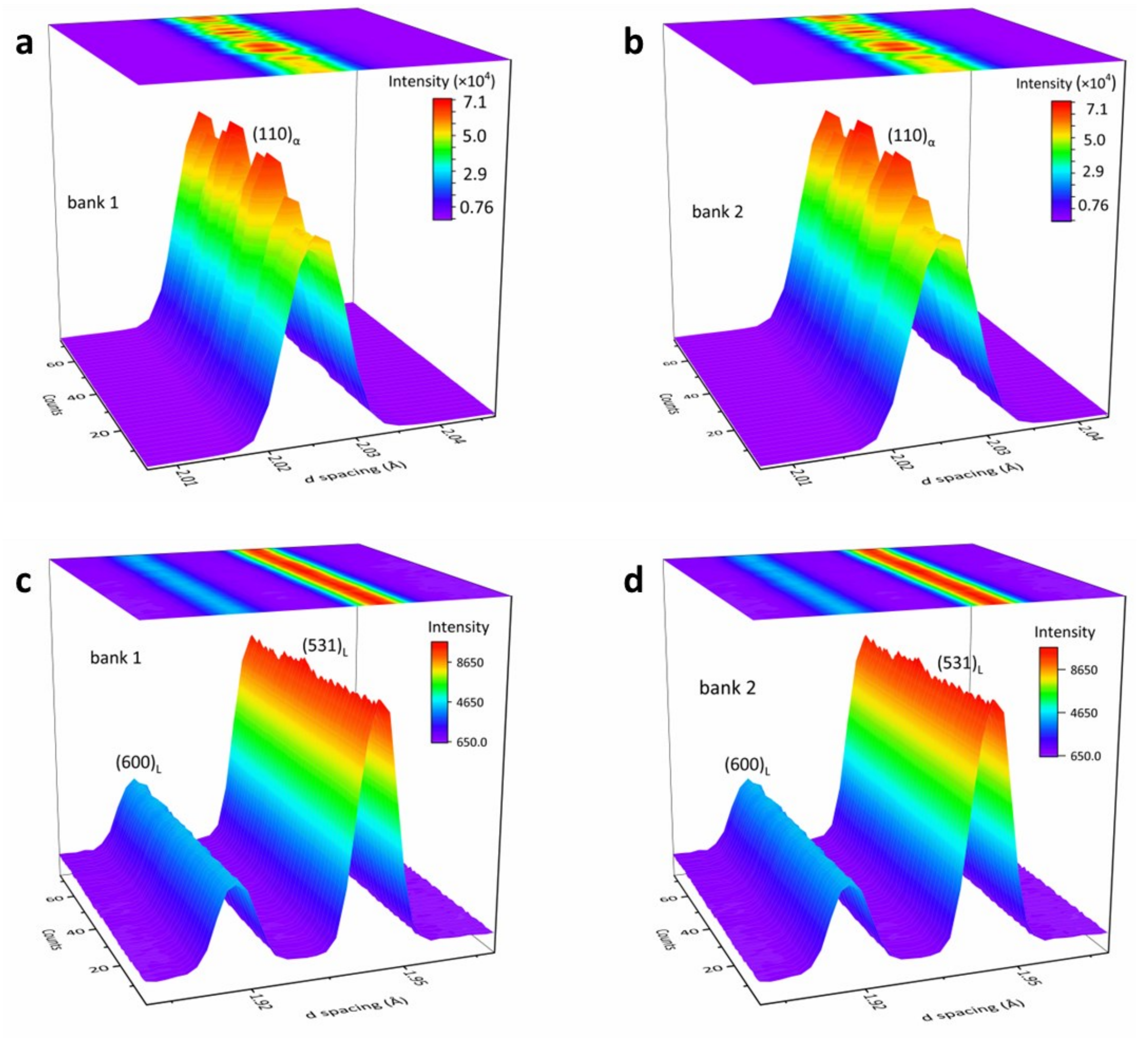

Supplementary Fig. 6 | The typical diffraction (*hkl*) reflections of the dual-phase alloy. a-b, The $(110)_{\alpha}$ reflection was collected by bank 1 and bank 2 detectors, respectively. **c-d**, The $(600)_{L}$ and $(531)_{L}$ reflections were collected by bank 1 and bank 2 detectors, respectively. The 75 counts are used to collect the three-dimensional crystallographic information (11.25 degrees/step for 45-degree vertical rotation; 24 degrees/step for 336-degree horizontal rotation). At each step, the two detectors will be collected, respectively.

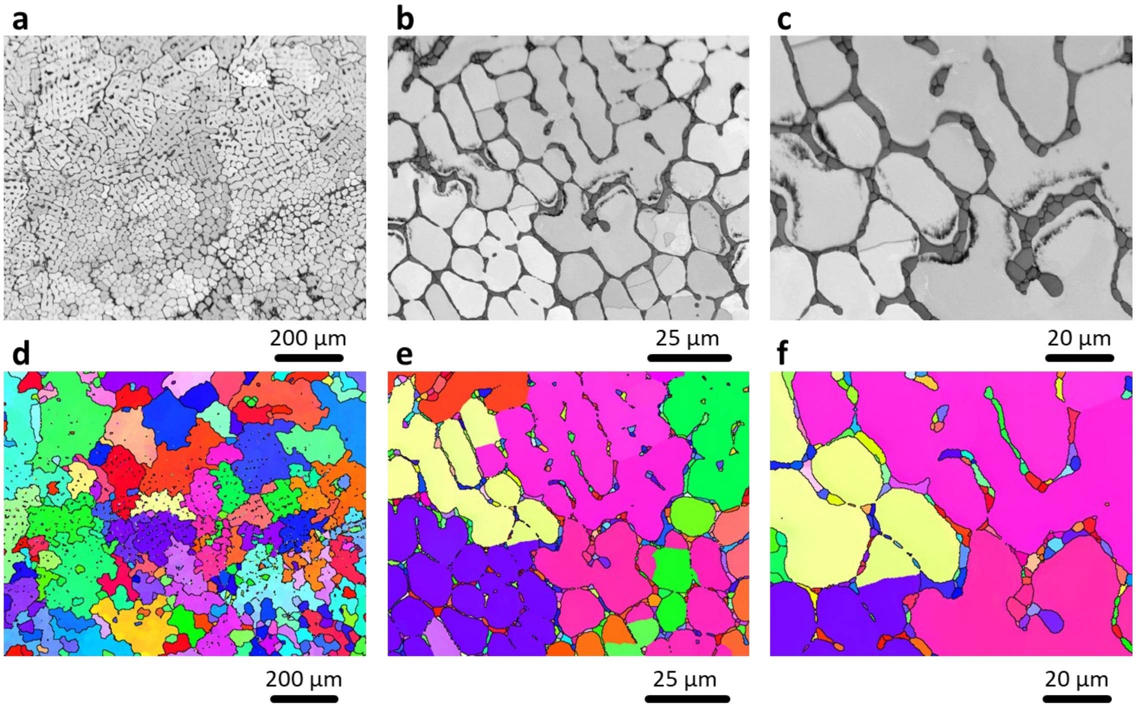

Supplementary Fig. 7 | The microstructure and inverse pole figure (IPFZ) of S-3 alloy. a-c, The EBSD band contrast figure at different magnifications. **d-f**, The EBSD inverse pole figure (IPFZ) at different magnifications.

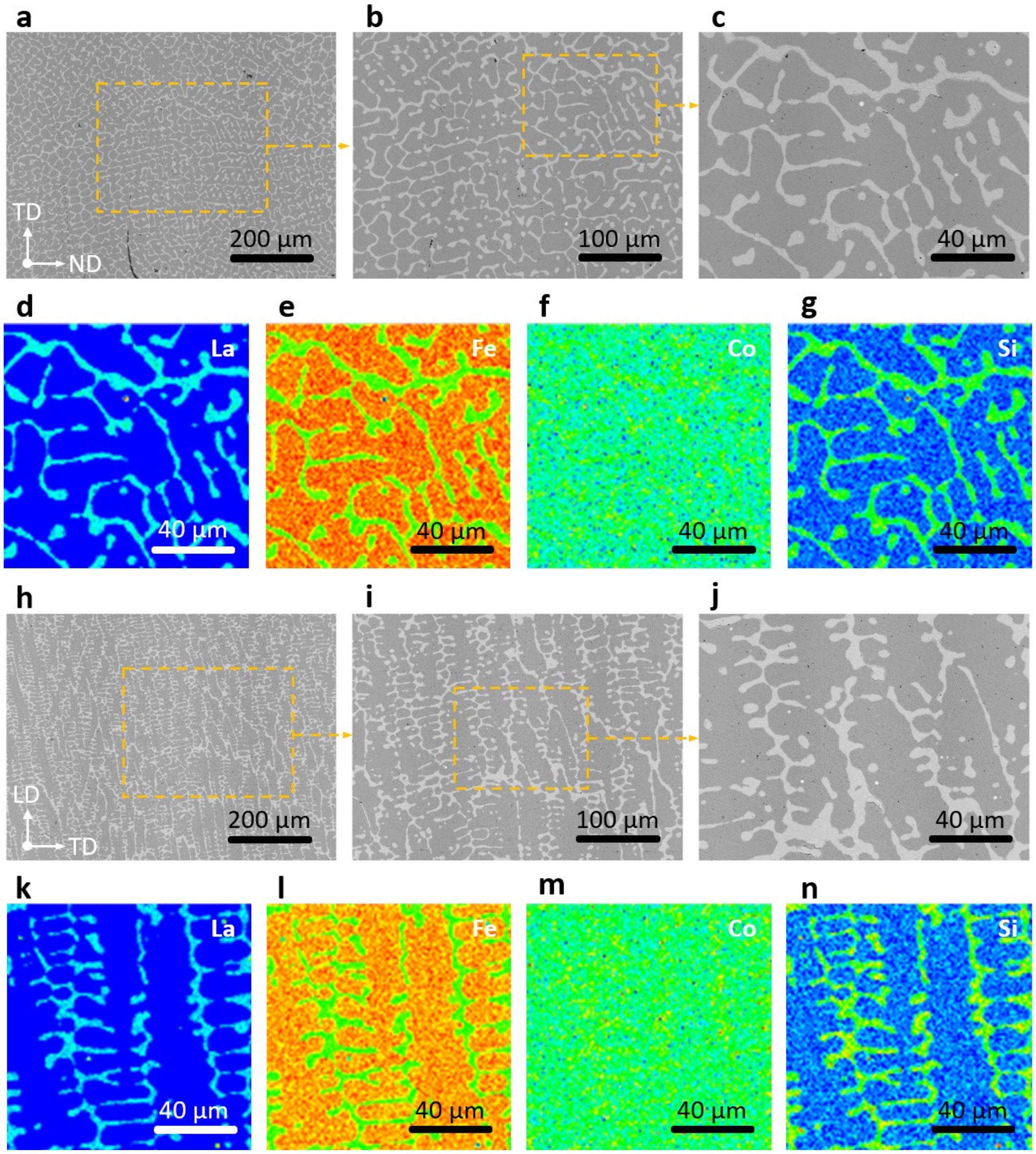

Supplementary Fig. 8 | The electro-probe micro-analyzer (EPMA) mappings of the S-3. a-c, The microstructure of S-3 in TD-ND plane at different magnifications. **d-g**, the elements mapping of La, Fe, Co, and Si, respectively. **h-j**, The microstructure of S-3 in LD-TD plane at different magnifications. **k-n**, the elements mapping of La, Fe, Co, and Si, respectively. We observed that there are certain differences in the morphology of the alloy (S-3) between the in-plane (TD-ND) and out-of-plane (LD-TD), the in-plane appearance is a continuous network and the out-of-plane shape is dendritic, which may be related to the growth direction of the as-cast alloy. As a result, the dilatometer thermal expansion behaves a negligible differences.

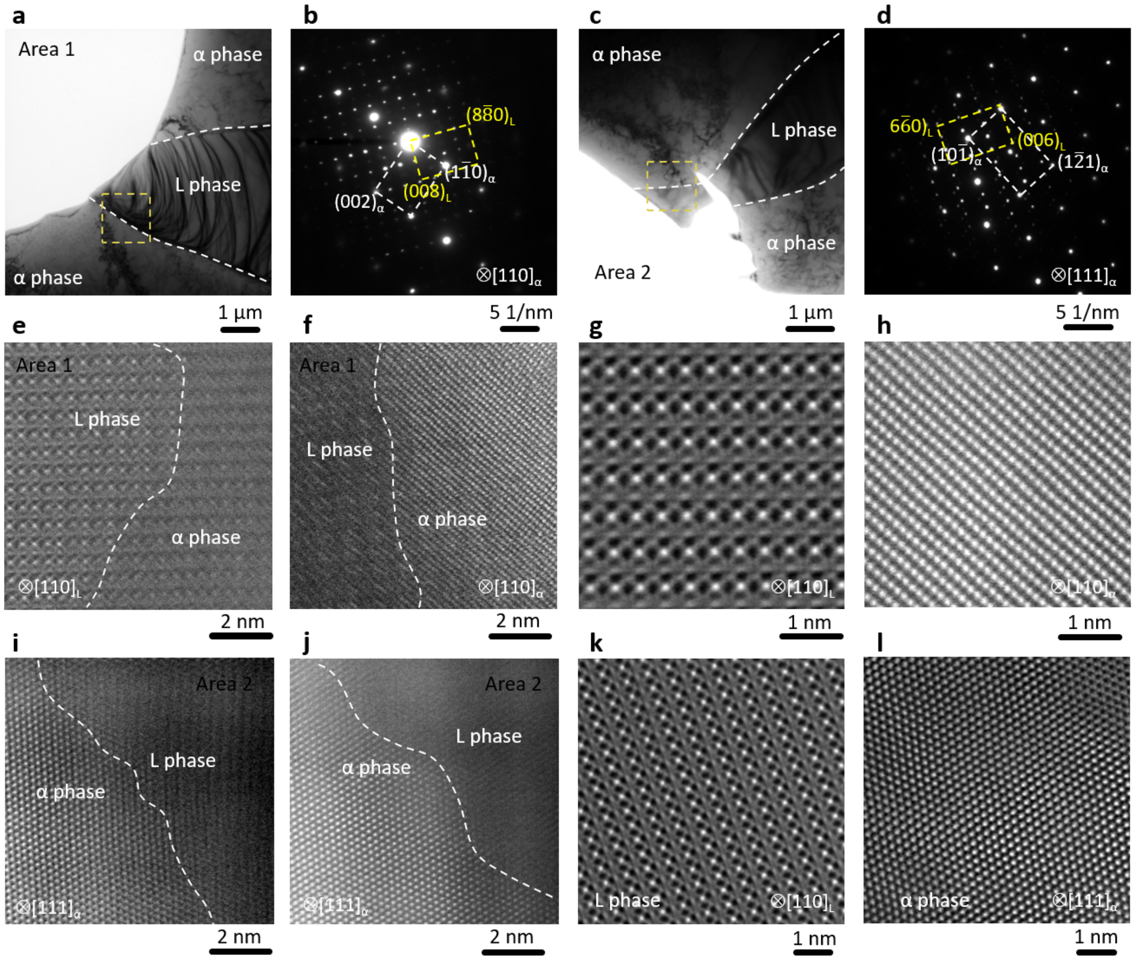

Supplementary Fig. 9 | The interface structure of the S-3alloy. a-d, The phase interfaces of the alloy and corresponding selected area electron diffraction (SAED). **e-l**, The HAADF-STEM images at the phase interface.

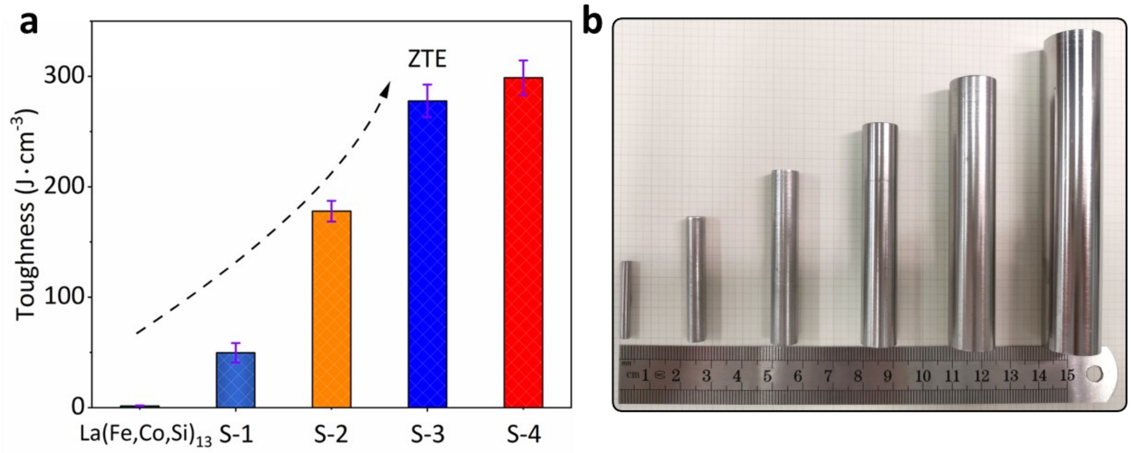

Supplementary Fig. 10 | Mechanical performance and small parts. a, Toughness comparison of S-1, S-2, S-3, and S-4 series alloys and targeted synthesis of the L phase. **b**, The super-high toughness S-3 alloy is facile and precisely to be machined. The rods/tubes are melted under induction melting conditions and cast into Φ 25 mm diameter rods, annealed under vacuum (1373 K + 24 hours), then machined to a suitable size and surface polished.

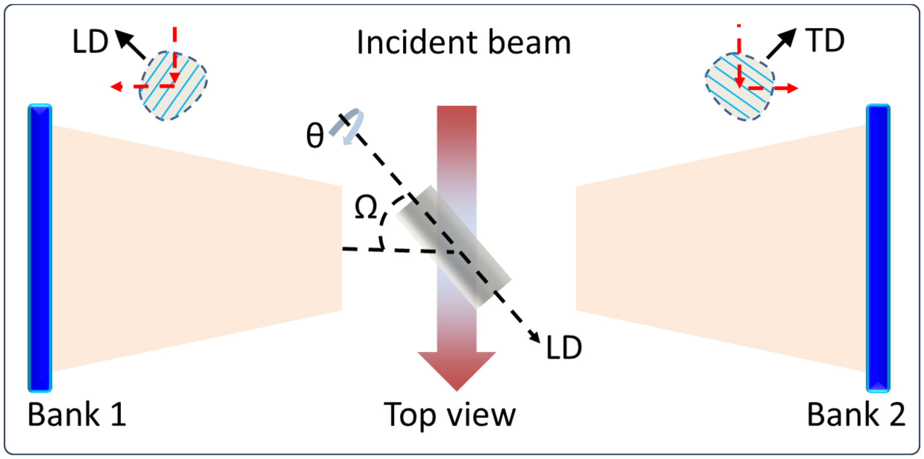

Supplementary Fig. 11 | Schematic diagram of the in-situ neutron diffraction experimental set-up from the top view. The sample is horizontal and positioned at 45° from the incident beam such that Bank 1 probes the strain component along the LD, while Bank 2 simultaneously probes the strain component in the TD, as shown in two insets.

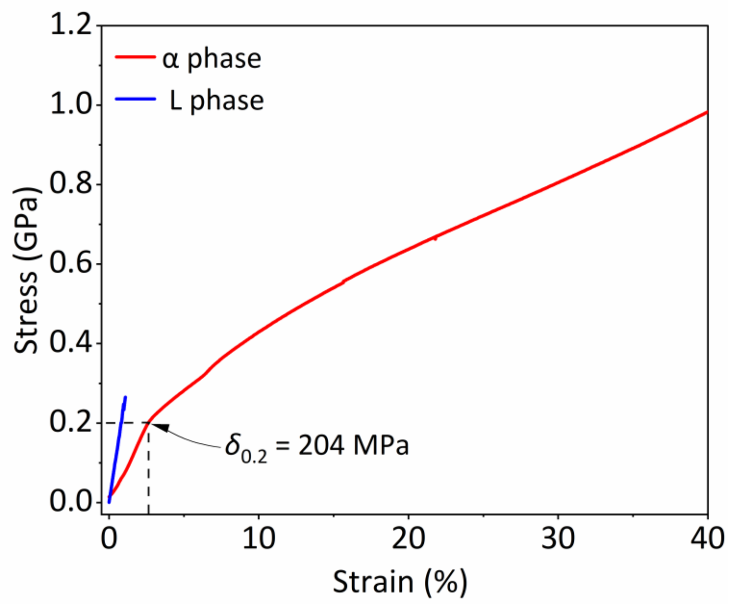

Supplementary Fig. 12 | The compressive stress-strain curves of the pure L phase ($LaFe_{10.30}Co_{0.83}Si_{1.87}$) and α phase ($Fe_{92.42}Co_{4.34}Si_{3.07}$). The L phase shows brittle but negative thermal expansion, and the α phase behaves as plastic and has positive negative thermal expansion.

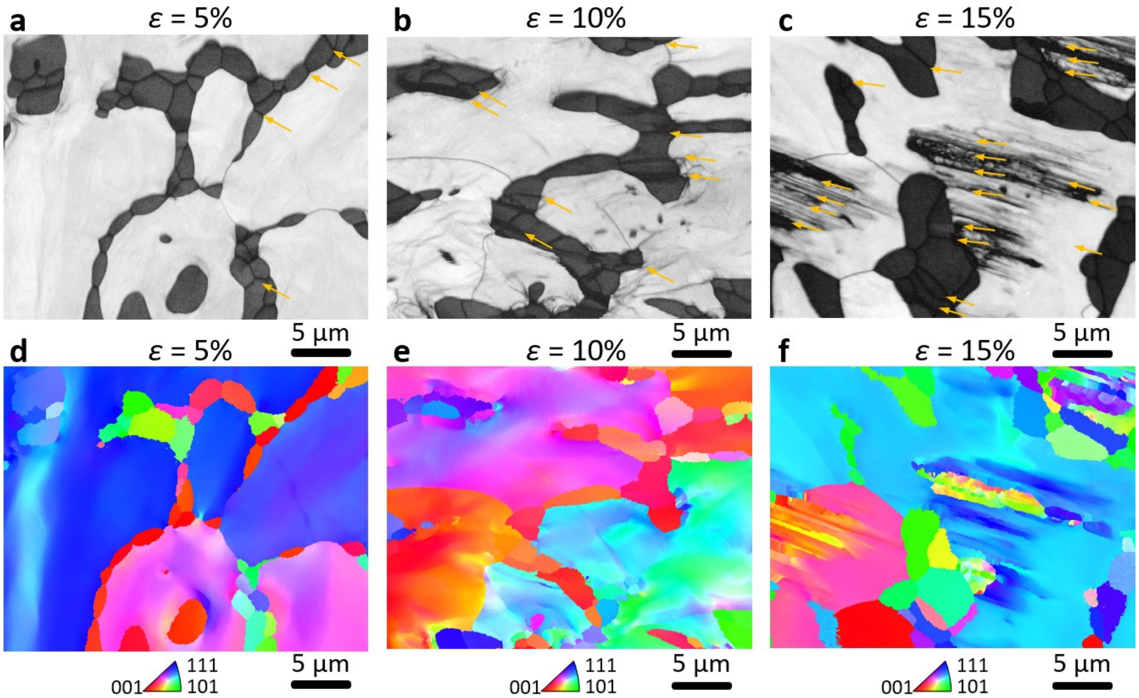

Supplementary Fig. 13 | Microstructure evolution of the dual-phase alloy (ZTE composition). **a-c,** Ex-situ EBSD phase contrast image under 5%, 10%, 15% strain (ε) conditions. Orange arrows represent shear cracks. **d-f,** Ex situ EBSD IPF inverse pole image under 5%, 10%, 15% strain (ε) conditions.

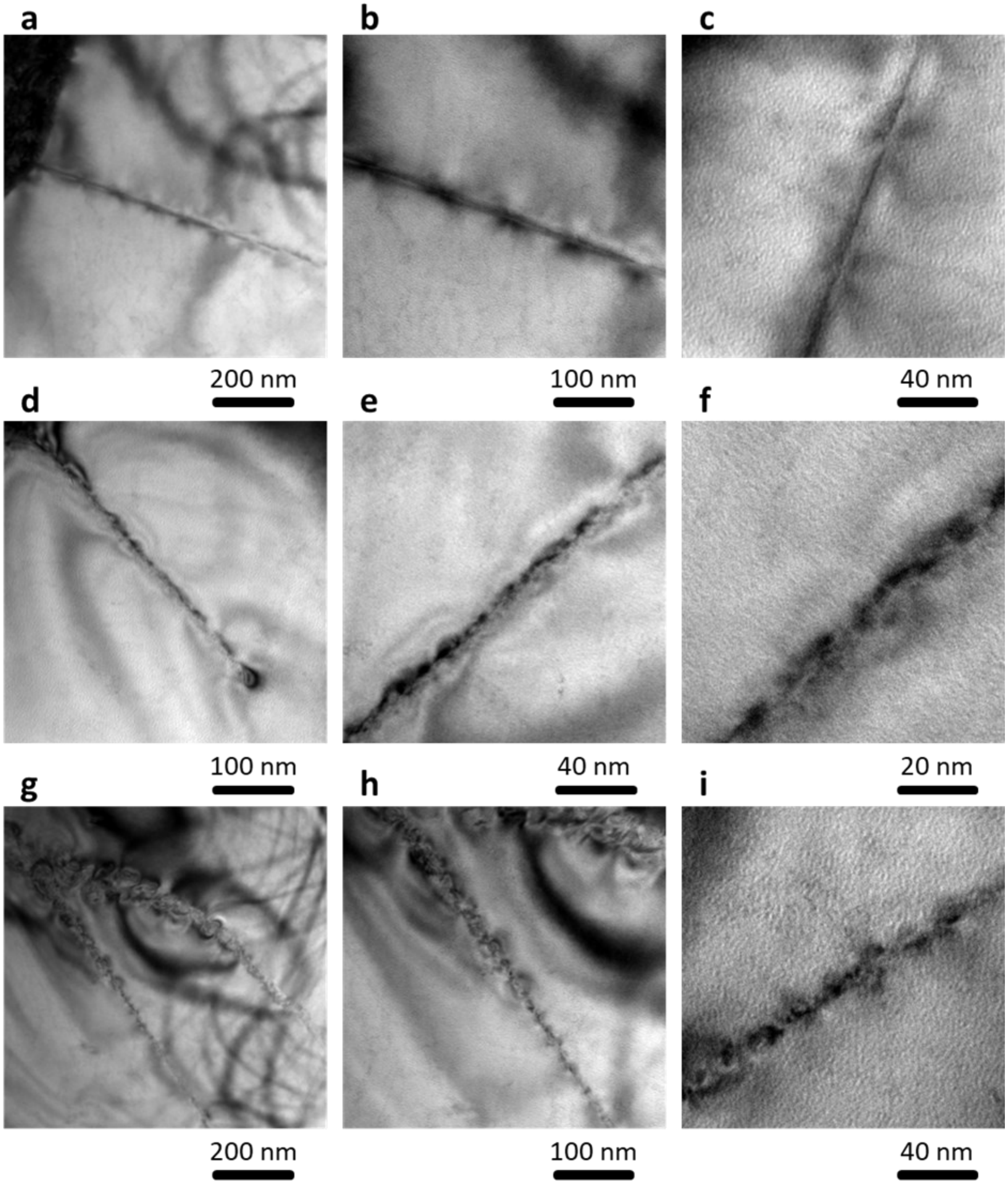

Supplementary Fig. 14 | Microstructure evolution of the dual-phase alloy (ZTE composition). **a-c,** Different stacking faults observed in the L phase at 5% strain (ε). This indicates that stacking faults are the main deformation mechanism in the initial stage of L-phase plastic deformation. **d-f,** Dual-phase interface structures at 15% strain (ε). The microcracks in the L phase are hindered from propagating to the interface.

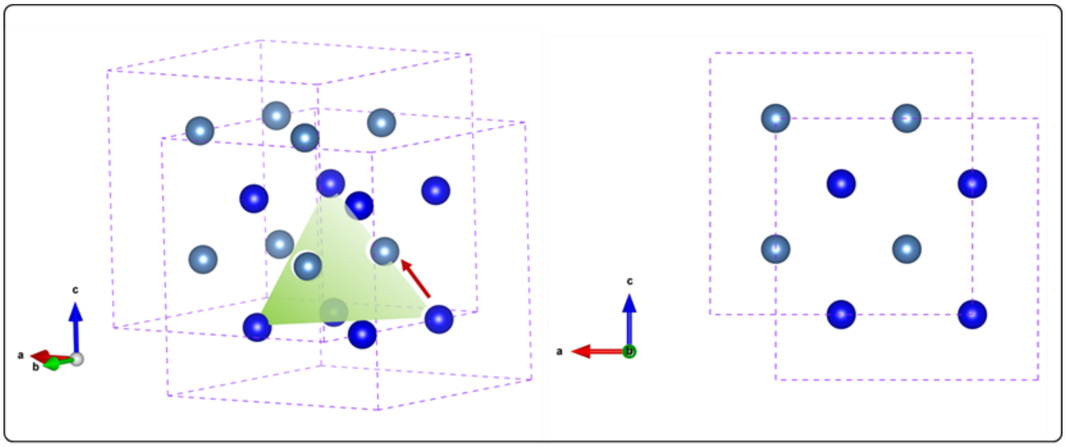

Supplementary Fig. 15 | The evolution of stacking fault illustrated by the crystal structure. To make the model clearer, we only keep La atoms here.

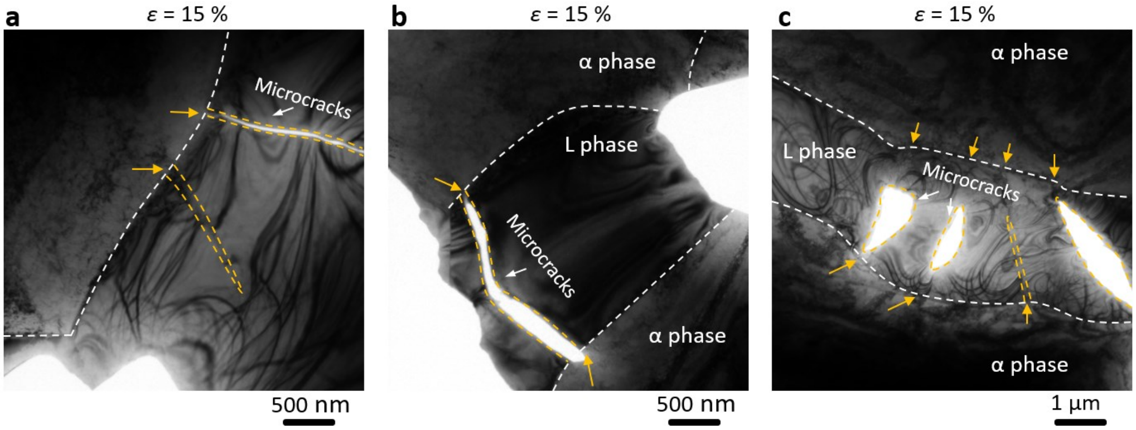

Supplementary Fig. 16 | a-c, Dual-phase interface structures at 15% strain (ε). The microcracks in the L phase are hindered from propagating to the interface. Noted: The cracks were affected by the thinning process. Noted: The structure of the cracks is affected to varying degrees by the thinning process.

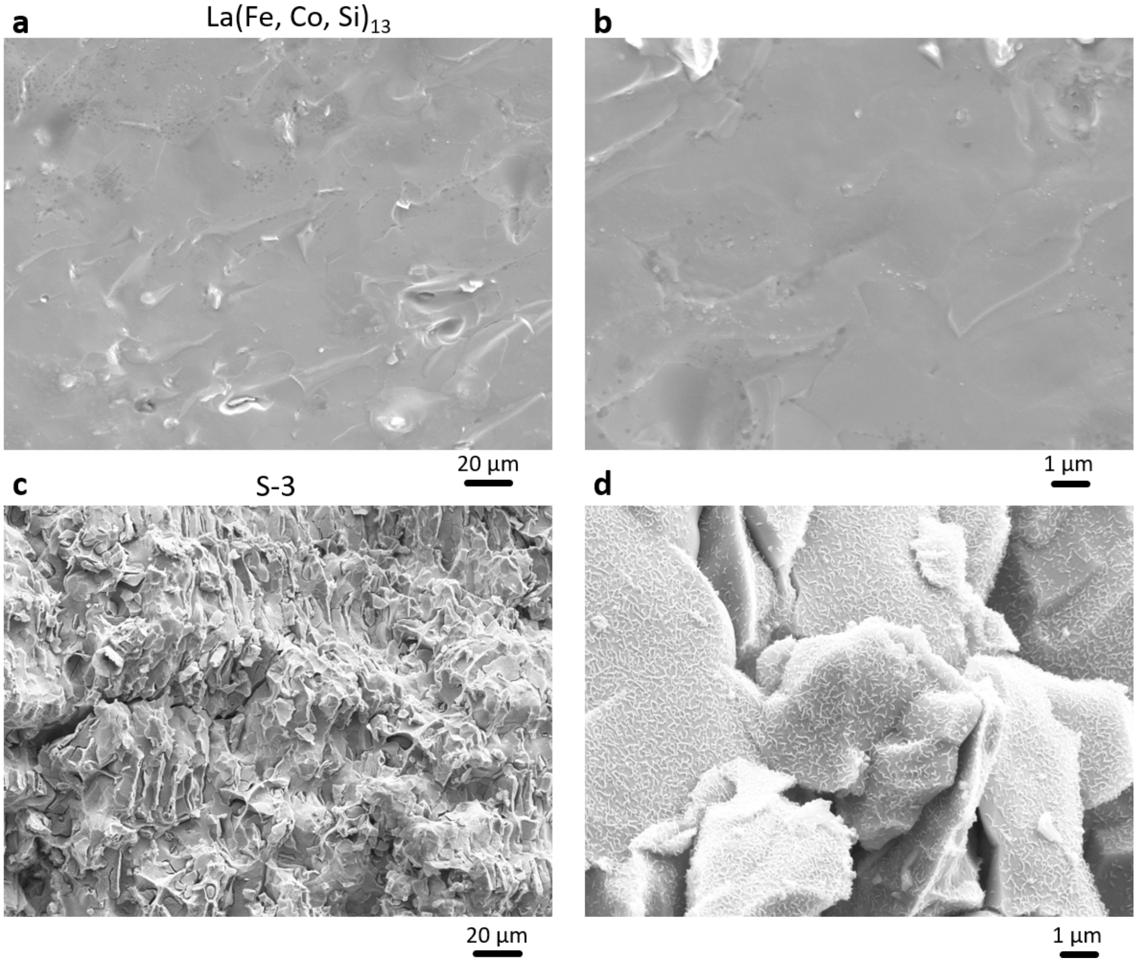

Supplementary Fig. 17 | a-d, Fracture morphology of the S-3 and pure La(Fe, Co, $Si)_{13}$ compound, dense and cross stripes demonstrated the large plastic deformation before failure.

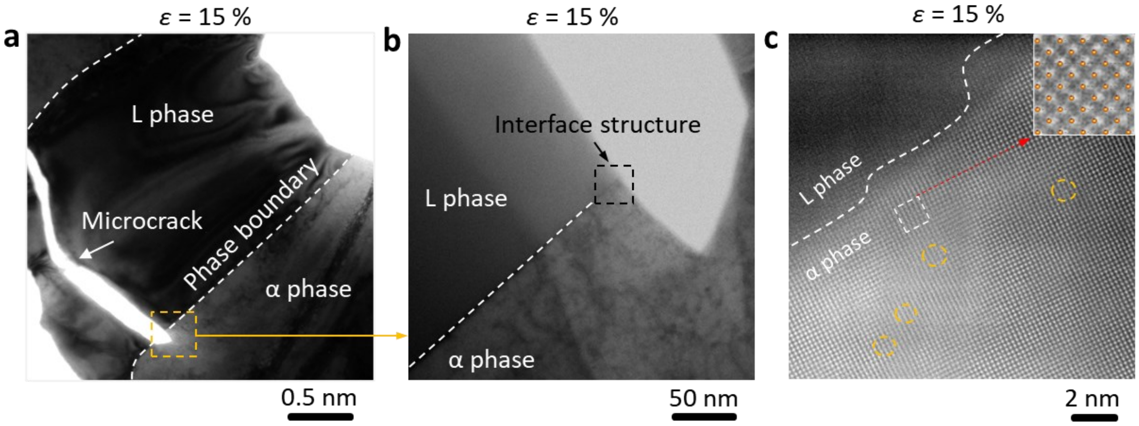

Supplementary Fig. 18 | A typical interface of the dual-phase alloy (ZTE composition) at 15 % strain (ε). a-c, The interface structure of the dual-phase alloy is determined by TEM at different scales.

Supplementary References

1. Song, Y., *et al.* Zero thermal expansion in magnetic and metallic Tb(Co,Fe)$_2$ intermetallic compounds. *J. Am. Chem. Soc.* **140**, 602-605 (2018).
2. Dan, S., *et al.* Zero thermal expansion with high Curie temperature in $Ho_2Fe_{16}Cr$ alloy. *RSC Adv.* **6**, 94809-94814 (2016).
3. Zhao, Y., *et al.* Giant negative thermal expansion in bonded MnCoGe-based compounds with Ni_2In-type hexagonal structure. *J. Am. Chem. Soc.* **137**, 1746-1749 (2015).
4. Li, S., *et al.* Zero thermal expansion achieved by an electrolytic hydriding method in La(Fe,Si)$_{13}$ Compounds. *Adv. Funct. Mater.* **27**, 1604195 (2017).
5. Song, X., *et al.* Adjustable zero thermal expansion in antiperovskite manganese nitride. *Adv. Mater.* **23**, 4690-4694 (2011).
6. Li, L., *et al.* Good comprehensive performance of Laves phase $Hf_{1-x}Ta_xFe_2$ as negative thermal expansion materials. *Acta Mater.* **161**, 258-265 (2018).
7. Song, Y., *et al.* Opposite thermal expansion in isostructural noncollinear antiferromagnetic compounds of Mn_3A (A = Ge and Sn). *Chem. Mater.* **30**, 6236-6241 (2018).
8. Yu, C., *et al.* Plastic and low-cost axial zero thermal expansion alloy by a natural dual-phase composite. *Nat. Commun.* **12**, 4701 (2021).
9. Hu, J., *et al.* Adjustable magnetic phase transition inducing unusual zero thermal expansion in cubic RCo_2-based intermetallic compounds (R = Rare Earth). *Inorg. Chem.* **58**, 5401-5405 (2019).
10. Wang, J., *et al.* Balancing negative and positive thermal expansion effect in dual-phase La(Fe,Si)$_{13}$/α-Fe in-situ composite with improved compressive strength. *J. Alloy Compd.* **769**, 233-238 (2018).
11. Liu, J., *et al.* Realization of zero thermal expansion in La(Fe,Si)$_{13}$ -based system with high mechanical stability. *Mater. Design.* **148**, 71-77 (2018).
12. Pang, X., *et al.* Design of zero thermal expansion and high thermal conductivity in machinable *x*LFCS/Cu metal matrix composites. *Compos. Part B-Eng.* **238**, 109883 (2022).
13. Zhou, H., *et al.* Tunable negative thermal expansion in La(Fe, Si)$_{13}$/resin composites with high mechanical property and long-term cycle stability. *Microst.* **2**, 1-15 (2022).
14. Cao, Y., *et al.* Ultrawide temperature range super-Invar behavior of R_2(Fe,Co)$_{17}$ materials (R = Rare Earth). *Phys. Rev. Lett.* **127**, 055501 (2021).
15. Lin, K., *et al.* High performance and low thermal expansion in Er-Fe-V-Mo dual-phase alloys. *Acta Mater.* **198**, 271-280 (2020).
16. Huang, R., *et al.* Giant negative thermal expansion in $NaZn_{13}$-type La(Fe, Si, Co)$_{13}$ compounds. *J. Am. Chem. Soc.* **135**, 11469-11472 (2013).
